# Factors Associated with Worse Lung Function in Cystic Fibrosis Patients with Persistent *Staphylococcus aureus*

**DOI:** 10.1371/journal.pone.0166220

**Published:** 2016-11-18

**Authors:** Sibylle Junge, Dennis Görlich, Martijn den Reijer, Bärbel Wiedemann, Burkhard Tümmler, Helmut Ellemunter, Angelika Dübbers, Peter Küster, Manfred Ballmann, Cordula Koerner-Rettberg, Jörg Große-Onnebrink, Eberhardt Heuer, Wolfgang Sextro, Jochen G. Mainz, Jutta Hammermann, Joachim Riethmüller, Ute Graepler-Mainka, Doris Staab, Bettina Wollschläger, Rüdiger Szczepanski, Antje Schuster, Friedrich-Karl Tegtmeyer, Sivagurunathan Sutharsan, Alexandra Wald, Jerzy-Roch Nofer, Willem van Wamel, Karsten Becker, Georg Peters, Barbara C. Kahl

**Affiliations:** 1 Clinic for Paediatric Pulmonology, Allergology and Neonatology, Hannover, Hannover, Germany; 2 Institute of Biostatistics and Clinical Research, University Hospital Münster, Münster, Germany; 3 Department of Medical Microbiology and Infectious Diseases, Erasmus Medical Center Rotterdam, Rotterdam, The Netherlands; 4 Institute of Medical Informatics and Biometrics, Technical University Dresden, Dresden, Germany; 5 Clinical Research Group, Clinic for Paediatric Pulmonology, Allergology and Neonatology, Medizinische Hochschule Hannover, Hannover, Germany; 6 CF-Center Innsbruck, Department of Child and Adolescent Health, Medical University of Innsbruck, Innsbruck, Austria; 7 Department of Paediatrics, University Hospital Münster, Münster, Germany; 8 Department of Paediatrics, Clemenshospital Münster, Münster, Germany; 9 Ruhr University, Paediatric Clinic at St Josef Hospital, Bochum, Germany; 10 Department of Paediatrics, University Hospital Essen, Essen, Germany; 11 Paediatricians “Kinderärztliche Ambulanz”, Hamburg, Germany; 12 CF Center, Department of Paediatrics, Jena University Hospital, Jena, Germany; 13 Department of Paediatrics, University Clinics Dresden, Dresden, Germany; 14 Department of Pediatrics, University Clinics Tübingen, Tübingen, Germany; 15 Department of Paediatric Pulmonology and Immunology, Charité Universitätsmedizin Berlin, Campus Virchow Klinikum, Berlin, Germany; 16 University Hospital Halle, Halle, Germany; 17 Children’s Hospital Osnabrück, Osnabrück, Germany; 18 Department of Paediatrics, University of Düsseldorf, Düsseldorf, Germany; 19 Park Schönefeld Clinics, Kassel, Germany; 20 Ruhrlandklinik, Essen, Germany; 21 University Clinics Leipzig, Leipzig, Germany; 22 Center for Laboratory Medicine, University Hospital Münster, Münster, Germany; 23 Institute of Medical Microbiology, University Hospital Münster, Münster, Germany; Laurentian, CANADA

## Abstract

**Background:**

*Staphylococcus aureus* is an important pathogen in cystic fibrosis (CF). However, it is not clear which factors are associated with worse lung function in patients with persistent *S*. *aureus* airway cultures. Our main hypothesis was that patients with high *S*. *aureus* density in their respiratory specimens would more likely experience worsening of their lung disease than patients with low bacterial loads.

**Methods:**

Therefore, we conducted an observational prospective longitudinal multi-center study and assessed the association between lung function and *S*. *aureus* bacterial density in respiratory samples, co-infection with other CF-pathogens, nasal *S*. *aureus* carriage, clinical status, antibiotic therapy, IL-6- and IgG-levels against *S*. *aureus* virulence factors.

**Results:**

195 patients from 17 centers were followed; each patient had an average of 7 visits. Data were analyzed using descriptive statistics and generalized linear mixed models. Our main hypothesis was only supported for patients providing throat specimens indicating that patients with higher density experienced a steeper lung function decline (p<0.001). Patients with exacerbations (n = 60), *S*. *aureus* small-colony variants (SCVs, n = 84) and co-infection with *Stenotrophomonas maltophilia* (n = 44) had worse lung function (p = 0.0068; p = 0.0011; p = 0.0103). Patients with SCVs were older (p = 0.0066) and more often treated with trimethoprim/sulfamethoxazole (p = 0.0078). IL-6 levels positively correlated with decreased lung function (p<0.001), *S*. *aureus* density in sputa (p = 0.0016), SCVs (p = 0.0209), exacerbations (p = 0.0041) and co-infections with *S*. *maltophilia* (p = 0.0195) or *A*. *fumigatus* (p = 0.0496).

**Conclusions:**

In CF-patients with chronic *S*. *aureus* cultures, independent risk factors for worse lung function are high bacterial density in throat cultures, exacerbations, elevated IL-6 levels, presence of *S*. *aureus* SCVs and co-infection with *S*. *maltophilia*.

**Trial Registration:**

ClinicalTrials.gov NCT00669760

## Introduction

Cystic fibrosis (CF) patients suffer from recurrent bacterial airway infections, which often lead to respiratory insufficiency and reduced life expectancy [1]. *Staphylococcus aureus* is one of the most frequently isolated pathogens from the airways of these patients [2–4]. Recent studies have shown the importance of *S*. *aureus* in young children with CF by way of eliciting an increased inflammatory response measured in broncho-alveolar lavages [5–9]. Thirty percent of the general population are persistent nasal carriers of *S*. *aureus* and this percentage is even higher in CF patients [10–12]. Nasal carriers have been shown to have a higher risk for *S*. *aureus* infections, but a lower rate of lethal infections [13]. It is often difficult to decide whether culturing *S*. *aureus* in respiratory specimens from CF patients is merely caused by colonization or whether this is also associated with worsening lung disease requiring antibiotic treatment. To support clinical decision, interleukin-6 levels in blood, which have been shown to increase in CF patients during airway inflammation [14], may serve as a potential marker for severity of inflammation.

In the present study, our goal was to determine risk factors associated with worse lung function in CF patients older than six years, who had been persistently colonized by *S*. *aureus*. We thus conducted a prospective observational longitudinal multi-center study (ClinicalTrials.gov: NCT00669760). We hypothesized that patients with high bacterial loads of *S*. *aureus* in airway specimens were more likely to experience worse lung function compared to patients with lower bacterial loads. Secondary objectives were the association between lung function and *S*. *aureus* nasal carriage in CF patients, the role of blood IL-6 levels and IgG levels against 44 *S*. *aureus* virulence factors, presence of small colony variants (SCVs) of *S*. *aureus* and co-infection with other CF-related pathogens such as *Stenotrophomonas maltophilia*, methicillin-resistant *S*. *aureus* (MRSA), *Aspergillus fumigatus*, *Achromobacter xylosoxidans* and *Mycobacterium abscessus* on lung function.

## Materials and Methods

### Study design

We conducted an observational prospective multicenter study to evaluate risk factors for worse lung function in patients with persistent *S*. *aureus* airway cultures older than 6 years. The treating physicians recruited CF patients for the study by using the following definitions for persistence from the medical records: 1. Patients presented with at least 2 positive cultures 6 months apart within one year before recruitment, or 2. half of the respiratory samples the year before recruitment were *S*. *aureus* positiv. The first patient was recruited in June 2008, the last patient finished the observation period in February 2010. At each visit throat or sputum cultures were obtained and sent to the central study laboratory located in Münster, Germany. On the basis of a study investigating chronic bronchitis patients [15], we hypothesized that CF-patients with high loads (in sputa ≥1x10^6^CFU/ml; in throat cultures semi-quantitative score ≥3) of *S*. *aureus* in their airway specimens are more likely to experience worsening of lung disease due to *S*. *aureus* than patients with low bacterial loads. To rule out an impact of *P*. *aeruginosa* or *Burkholderia cepacia* complex (BCC) on lung function, patients with cultures of these two pathogens were excluded. We defined exacerbation by evaluating clinical symptoms established by Fuchs et al. for the assessment of exacerbation [16]. If a patient met at least four out of 11 evaluated symptoms, the clinical status was determined as exacerbation.

Secondary objectives included the association between nasal *S*. *aureus* carriage, the level of IL-6, the presence of *S*. *aureus* small colony variants (SCVs) and the co-culture of other important CF pathogens such as *S*. *maltophilia*, *A*. *xylosoxidans*, *A*. *fumigatus* and *M*. *abscessu*s and the risk for exacerbation or worse lung function.

#### Inclusion criteria

CF Individuals older than six years were included with persistent *S*. *aureus* airway cultures one year before recruitment.

#### Enrollment and follow-up

Patients identified as persistently colonized by *S*. *aureus* as defined by our inclusion criteria were enrolled in this study by their attending CF specialists. If *P*. *aeruginosa* or BCC were cultured for more than 6 months during the study period, patients were excluded from further analysis. Patients were followed for a period of 21 months with regular visits (usually every 3 months). Sputum samples were spontaneously produced. There were no requirements for induced sputum cultures. Blood samples were taken from patients once yearly and/or at exacerbations. An ethical statement was obtained at the main study center with the central laboratory in Münster, Germany (2007-496-f-S). Other ethical statements have been obtained from the Universitätsklinikum Tübingen 450/2008B02, Martin-Luther-Universität Halle Wittenberg hm/bu, Ärztekammer Hamburg MC039/09 and the Universitätsklinikum Dresden. All other ethic committees at the Ruhr Universität Bochum, Universitätsklinik Essen, Medizinische Hochschule Hannover, Universitätsklinikum Jena, Charité Universitätsmedizin Berlin, Campus Virchow Klinikum, Kinderkrankenhaus Osnabrück, Universitätsklinik Innsbruck, Universitätsklinik Düsseldorf, Park Schönefeld Klinik Kassel and the Universitätsklinikum Leipzig waived their approval. Written informed consent was obtained from all patients and parents, if patients were younger than 18 years. Clinical trial registered with www.clinicaltrials.gov (NCT00669760).

### Investigated Fuchs criteria and clinical report forms (CRFs) [16]

At every visit patients performed lung function tests assessed as forced expiratory volume in 1 second (FEV_1_). Other recorded clinical parameters included the presence of fatigue, malaise or lethargy, sinus discharge, cough and hemoptysis as well as sputum volume or color. These data were documented by the treating physicians in CRFs, together with *CFTR* genotype, pancreas sufficiency/insufficiency, physical and radiographic findings on examination of the chest, body mass index and antibiotic therapy. The CRFs were sent together with the respiratory specimens to the central laboratory in Münster, Germany.

### Microbiology

All airway and blood samples obtained from patients were sent to the central study laboratory (Medical Microbiology, Münster, Germany) within 24 hours and were cultured according to standard procedures for CF airway cultures [17,18]. All samples were streaked on Columbia sheep blood agar (Becton Dickinson, Heidelberg, Germany), MacConkey agar for Gram-negative bacteria (Becton Dickinson, Heidelberg, Germany), SAID chromogenic agar for *S*. *aureus*, (bioMerieux, Nürtingen, Germany), all incubated for 48h at 37°C; on chocolate agar (Mast Diagnostica, Reinfeld, Germany) for *Haemophilus influenzae*, incubated for 48h at 37°C with 5% CO_2_; on BCSA agar (bioMerieux, Nürtingen, Germany) for *Burkholderia cepacia complex*, incubated at 30°C for 10 days, and on Kimmig agar for fungi. In case of mucoid consistency, sputa were incubated with sputasol (dithiotreitol, Oxoid, Wesel, Germany) for 30 min at 37°C and homogenized by vortexing and vigorous pipetting before further processing. Quantitative cultures of sputa were performed by serial dilution of 500μl of sputum in 4.5ml 0.85% NaCl according to standard procedures. Semi-quantitative analysis was performed for throat and nasal cultures and for sputa of less than 500μl sputum. A score of 1 was assigned to single *S*. *aureus* colonies on the primary agar plate, a score of 2 to medium density and a score of 3 to highly dense numbers of *S*. *aureus* colonies on the primary agar plate, respectively. SCVs were identified by sub-culturing isolates of interest from Columbia blood and SAID agar on Columbia blood agar (incubated at 37°C) and on Schaedler agar (incubated at 37°C with 5% CO_2_) for 24h [19]. All isolates, which displayed small colony size on Columbia blood and normal size on Schaedler agar were identified as SCVs, which was further confirmed by a positive catalase and positive Pasteurex Staph Plus test (BIO-RAD, München, Germany). In case of negative results, isolates were identified by 16S-RNA sequencing.

### Measurements of anti-staphylococcal antibodies

The levels of IgG antibodies against 44 staphylococcal antigens in serum samples of CF patients and 53 healthy nasal carriers, who consisted of volunteers of the Institute of Medical Microbiology and medical students, were measured using a bead-based flow cytometry technique (xMAP^®^; Luminex Corporation) as previously described [20,21]. All examined antigens are given in S1 Supporting Material.

### IL-6 measurements

IL-6 levels in sera were determined by ELISA (R&D, Wiesbaden, Germany) according to manufacturer instructions and S1 Supporting Material.

### Statistical analysis

Patient data were collected longitudinally. The baseline patient characteristics were analyzed by standard descriptive statistics. Categorical variables were described by absolute and relative frequencies. Continuous variables were described by median and range. The following variables were computed:

Lung function (FEV_1_% predicted) was determined on the basis of Quanjer et al. [22]. SCV status (never/ever) of the patients was positive (ever), if SCVs were detected in specimens at any visit. Carrier status was deemed positive, if in at least at 50% of visits *S*. *aureus* nasal carriage was detected. Exacerbation status (never/ever) was calculated using the symptoms defined by Fuchs [16]. A visit with Fuchs score in excess of 4 was defined as an exacerbation visit. Patients exhibiting at least one exacerbation visit were assigned to the exacerbation ever group. *S*. *aureus* density [sputa: high (<10^6^CFU/ml and low <10^6^CFU/ml according to Mensa and Trilla for patients with chronic bronchitis;[15] nasal and throat swabs: high >/ = +++; low + or ++)] was determined based on quantitative and semi-quantitative data per visit. Patients’ co-infection status (never/ever) with MRSA, *S*. *maltophilia* and *A*. *fumigatus* was determined by compiling information from all visits.

Outcome parameters of interest were bacterial density (high/low) in different specimens, lung function (FEV_1_% predicted) and interleukin-6 (IL-6) levels.

Cross-sectional analyses, e.g. at baseline, were performed using Mann-Whitney-U tests from continuous variables and Chi-Squared tests, or Fisher’s exact tests for categorical variables. Longitudinal analyses were performed to assess the effect of different explanatory variables on the outcome parameters. To model patient progress over time we used generalized linear mixed models (GLMM). Bacterial density was modeled by a binary distribution. FEV_1_% proved to be normally distributed. IL-6 levels were skewed to the right and a log-normal distribution was used to fit the data. The canonical link function was used. For all models, a random effect for the individual progress of each patient over the study time (days from first visit) was included using sp(pow) as covariance structure. All models were age and gender adjusted, if not otherwise specified. Studentized residual plots were used to examine model requirements.

To analyze the functional dependency of FEV_1_% on IL-6 levels a polynomial model was used: *FEV*1 = *β*1 × [*IL*6]^*β*2^. The model coefficients were fitted using only baseline measurement using procnlin in SAS.

Each variable for the specific IgG measurements was tested for normal distribution using histogram plots, Q-Q plots and the Kolmogorov-Smirnov test. For the comparison of the IgG levels between patients and healthy controls the multiple testing problem was controlled by Bonferroni correction. Age and gender adjusted GLMM were used to analyse specific IgG-responses according to carrier status (yes/no), density (high/low) in sputa, SCV (ever/never) including a random effect for repeated measurement. q-values were Bonferroni–Holm adjusted p-values to control the FDR on the multiple significance level of 5%. IgG-levels were modelled as continuous factors. Odds ratios (OR) were interpreted as a factor, by which the risk for carriage/high-density/SCV was changed per 100 units in antigen levels. For an association of IL-6 and FEV_1_% predicted with IgG-levels, estimates were interpreted as mean change in IL-6 per 1 unit in IgG-levels.

The local significance level for all performed tests was α = 0.05. 95%-confidence intervals are given, where appropriate. Statistical tests are performed exploratory and can be interpreted as hypothesis generating. Statistical analysis was performed using SPSS (v. 22, IBM) and SAS (v. 9.4, SAS Institute, Cary, NC).

## Results

### Demographics and statistics

A total of 195 patients were recruited from 16 German centers and one CF center in Austria, which take care of 1980 CF patients. Thirteen of 195 patients r were excluded due to the chronic culture (> 6 months) of *P*. *aeruginosa* (n = 12) or *BCC* (n = 1) during the study period. Cystic fibrosis transmembrane regulator (*CFTR*) genotypes were reported for 173 of 195 patients, see Table 1. Patients with non-p.Phe508del *CFTR* genotypes (n = 48; 27.7%) were overrepresented compared to the German CF patient population (Table 1, p<0.0001). The median age of patients was 16 years (range 5.8 y– 42 y). A preponderance of male to female subjects compared to the gender ratio of the German CF registry was reported (Table 1, p<0.001).

**Table 1:** Characteristics of study patients compared to the German CF population.

	Study patients^a^	German CF patients	p-value
**# of patients**	195	4456	
**Age (in years)**	16 (6–42)	20 (6–70)	<0.001
**Male**	120 (61%)	2121 (48%)	<0.001
**Ex.panc.suff.**^b^	22 (11%)	1426 (32%)	<0.001
**FEV**_**1**_ **(% predicted)**	84.1 (13–121)	72.0 (10–145)	<0.001
**BMI-quantils**	25% (0.07–100%)	27% (0.05–100%)	0.007
**Genotype**^c^	173	3832	
**F508del homo**^d^	85 (49%)	1863 (49%)	<0.001
**F508del hetero**^e^	40 (23%)	1429 (37%)	
**others**	48 (28%)	540 (14%)	

^a^Age, FEV1, BMI and exacerbation numbers are reported as median (range), respectively. Exocrine pancreatic sufficiency and genotype are reported as absolute frequencies (relative frequencies). We did not exclude one patient, who was 5.8 years at the first visit, but was 6.1 years at the second visit with seven reported visits throughout the study period.

^b^exocrine pancreatic sufficiency

^c^*Cftr* genotype of patients available

^d^F508del homozygous

^e^F508del heterozygous

The mean number of all visits was seven (range 1–18). Clinical data of patients at baseline are shown and compared with national data concerning German CF patients in Table 1. Lung function decline was -1.2717 FEV_1_% predicted per years of age calculated for the study group at the first visit (CI -1.6676/-0.7235; p<0.0001).

### Age-bacterial density association

For 98 patients at least one sputum (n = 446, range 1–14) was available for analysis, while for 97 patients only throat cultures (n = 663, range 1–11) were available. The age of patients at the time of the first positive *S*. *aureus* sputum culture was significantly associated with *S*. *aureus* density in sputa, indicating that older patients had significantly higher bacterial loads in sputa compared to younger patients (p = 0.018). Patients with low bacterial density in sputa were on average 19.2 years of age, while patients with high bacterial loads were 22.2 years of age, respectively. There was no association between age and *S*. *aureus* density for patients with only throat (p = 0.7016) or nasal cultures (p = 0.411).

### Bacterial density and lung function

Bacterial density according to specimens is shown in Table 2. No significant association, adjusted for age and sex, was reported between *S*. *aureus* density and FEV_1_% predicted in neither patients with sputum (p = 0.5151) nor throat cultures (p = 0.185). There was also no association of FEV_1_% predicted with BMI-quantiles for both groups (p = 0.4352; p = 0.1007) neither at baseline nor during the study period.

**Table 2:** Positive airway specimens according to *S*. *aureus* density.

Specimens	Low density (<1x10^6^CFU/ml) score 1, 2^a^ (%)	High density (>/ = 1x10^6^CFU/ml) score 3^b^ (%)	Sum
**Nasal swabs**	517 (68.7)	236 (31.3)	753
**Nasal lavage**	26 (76.5)	8 (23.5)	34
**Throat swabs**	545 (69.7)	237 (30.3)	782
**Sputa**	166 (42.1)	228 (57.9)	394
**All**	1254 (63.9)	709 (36.1)	1963

^a^low bacterial density of sputum (<1x10^6^CFU/ml) and throat cultures (score 1 and 2)

^b^high bacterial density of sputum (≥1x10^6^ CFU/ml) and throat cultures (score ≥3)

**Fig 1:**
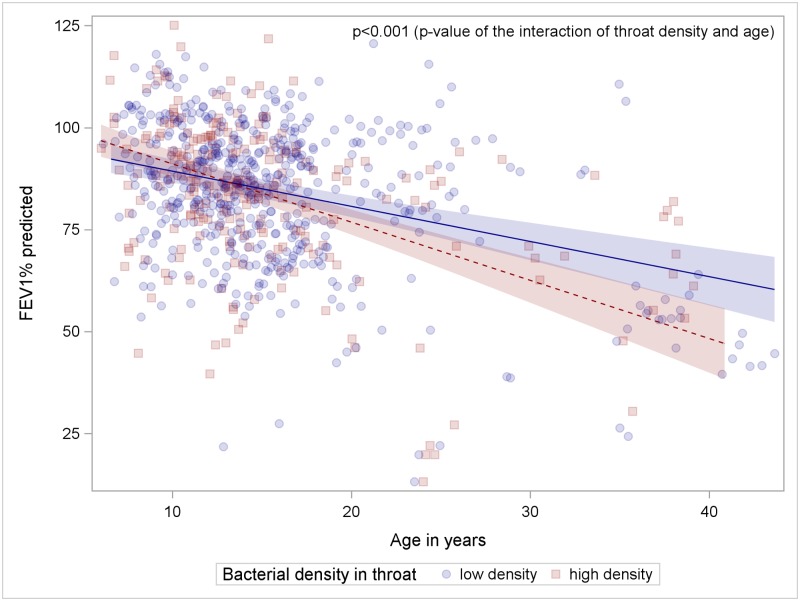
Patients with throat cultures with high bacterial density experience a more rapid lung function decline. Patients with high bacterial density in throat specimens are indicated by red squares, patients with low bacterial density in throat specimens by blue circles, the fitted model prediction is coloured accordingly.

However, bacterial density in throat specimens was significantly associated with annual lung function decline. Patients with high density lost 1.428% FEV_1_ per year compared to patients with low density, Fig 1 (0.8626%FEV_1_; p<0.001 of the interaction of age and density). We could not observe similar associations with respect to bacterial density in sputum cultures and lung function decline (data not shown).

**Fig 2:**
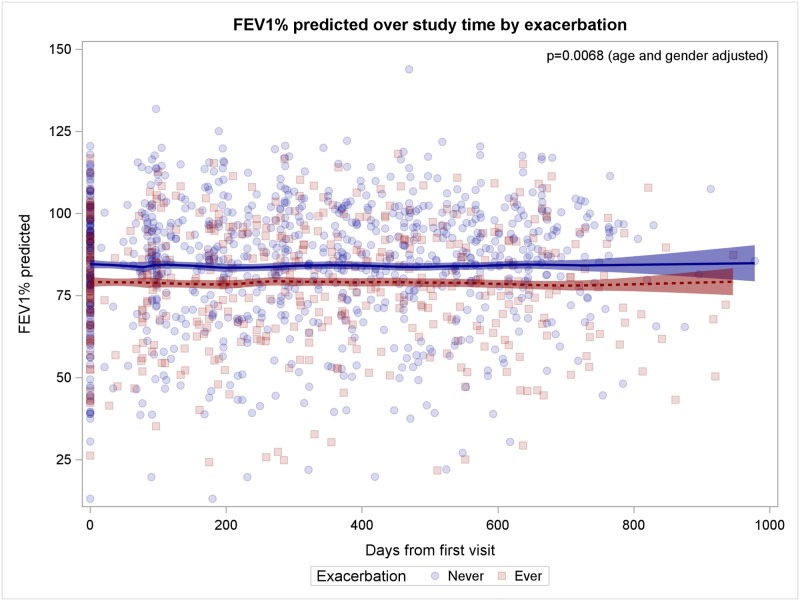
Patients with exacerbations have worse lung function. Patients with exacerbation are indicated by red squares, patients without exacerbation by blue circles, the fitted model prediction is coloured accordingly.

**Fig 3:**
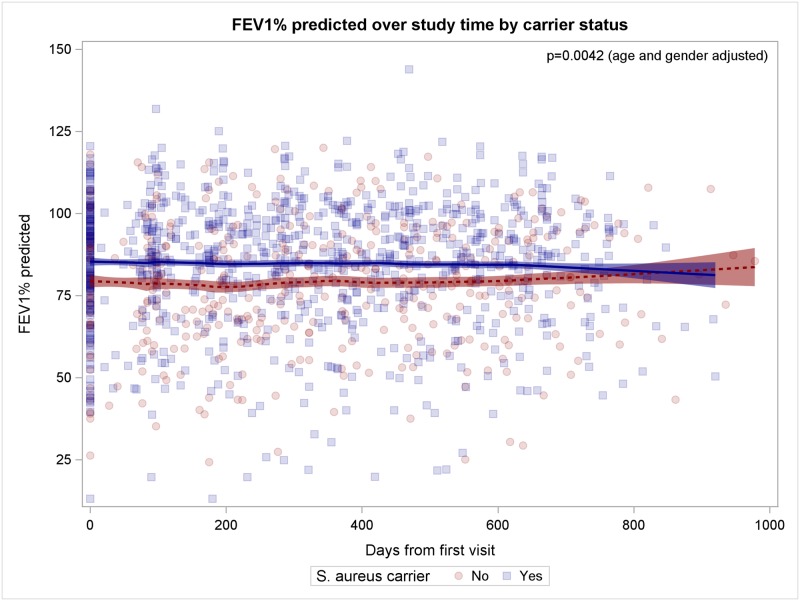
Patients with *S*. *aureus* nasal carriage have better lung function. *S*. *aureus* nasal carriers are indicated by blue squares, non-nasal carriers by red circles, fitted model prediction is coloured accordingly.

**Fig 4:**
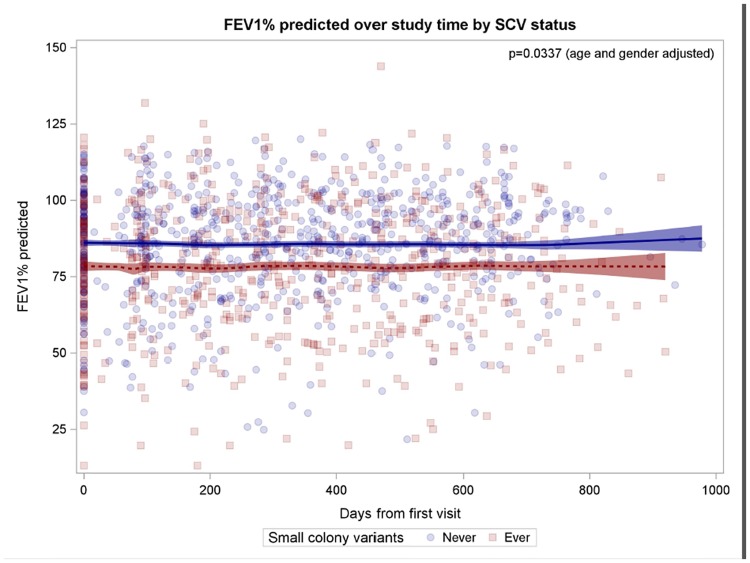
Patients with SCVs have worse lung function. Patients with SCVs are indicated by red squares, patients without SCVs by blue circles, fitted model prediction is coloured accordingly.

**Fig 5:**
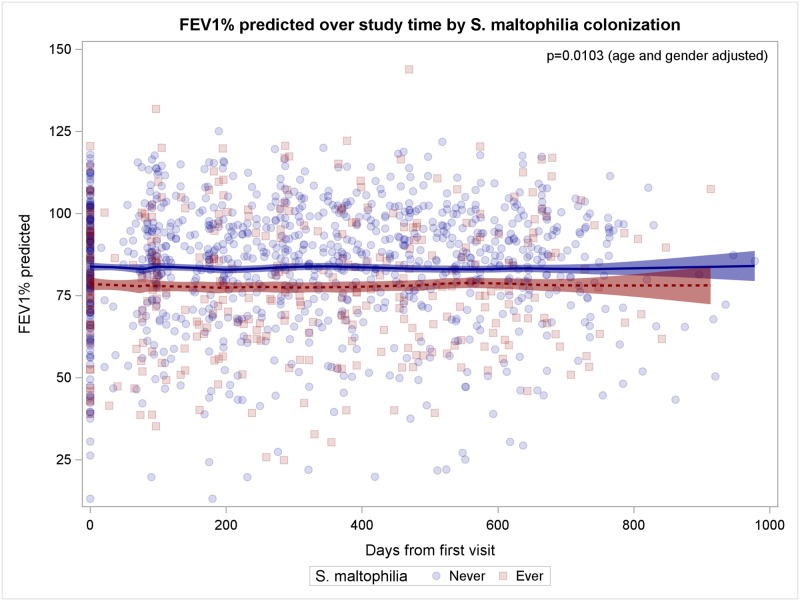
Patients with culture of *S*. *maltophilia* have worse lung function. Patients with *S*. *maltophilia* are indicated by red squares, patients without *S*. *maltophilia* by blue circles, fitted model prediction is coloured accordingly.

The Figs 1–5 show observed FEV_1_% predicted measurements over study time (days from first visit). Lines represent the LOESS-fit including 95% confidence intervals of the predicted values of the generalized linear mixed model.

There was a strong association between FEV_1_% predicted and bacterial density and cumulative clinical symptoms according to Fuchs criteria [16] for patients who expectorated sputa (95 patients, max. 9 visits; p = 0.0661, S1 Fig), but not for patients with throat cultures (167 patients, max. 9 visits).

### Patients with exacerbations have worse lung function

Sixty of 195 patients (31%; 55% male) experienced exacerbations with a mean number of 0.384 exacerbations per patient (range 0, 4). The age distribution of patients with and without exacerbation did not differ significantly (assessed at baseline, p = 0.5182, S1 Table). Using a longitudinal multivariable model adjusted for age and sex our data showed that patients with exacerbations have worse lung function of 5.40% FEV_1_% predicted (95%CI 1.50 to 9.30 FEV_1_%) less compared to patients without exacerbations (p = 0.0068, Fig 2). Patients with exacerbations have a lung function decline per year of age of 1.22% FEV_1_% predicted (95%CI 0.94 to 1.50 FEV_1_%) compared to 0.88% FEV_1_% (95%CI 0.64 to 1.12 FEV_1_%) predicted in patients without exacerbations (p = 0.0018). Patients with exacerbations received more antibiotics than patients without exacerbations (p = 0.037, OR = 1.175). For patients with exacerbations antibiotic treatment was reported at five visits (mean 4.3), while for patients without exacerbations at three visits (mean 3.43, p = 0.021), respectively.

### Patients with *S*. *aureus* nasal carriage

Since persistent nasal *S*. *aureus* carriers differ from non-carriers in terms of severe *S*. *aureus* infections [10], we determined the nasal carriage status of our study patients to analyze its impact on CF lung disease. CF patients with persistent nasal carriage (n = 122, 62.6%) were more likely male patients compared to patients without persistent nasal carriage (p = 0.00075, S1 Table) and had significantly more nasal cultures with high bacterial loads (p = 0.0255). *S*. *aureus* nasal carriers had better lung function throughout the study compared to non-carriers with a difference of 5.758 FEV_1_% predicted (95%CI 1.84 to 9.68; p = 0.0042; Fig 3). It is noteworthy that female nasal carriers experienced better lung function throughout the study period compared to male carriers (p = 0.0064, age-adjusted).

### Patients with *S*. *aureus* SCVs

To determine the impact of *S*. *aureus* SCVs on lung disease data were analyzed in relation to the culture of SCVs in airway specimens, which were cultured from 84 patients (43%) at least once. Patients with SCVs were significantly older (p = 0.0066), had lower FEV_1_% predicted at baseline (p = 0.0133) and during the study period (p = 0.0337, age and gender adjusted; lower lung function of 4.099 FEV_1_% predicted (95%CI 0.32 to 7.88 FEV_1_%) compared to patients without SCVs (Fig 4). Within a multivariable model also including antibiotic treatment, the association of SCVs with worse lung function was still significant (p = 0.0433, lower lung function in patients with SCVs of 3.89 FEV_1_% predicted, 95%CI 0.12 to 7.66 FEV_1_%). In addition, patients with SCVs were more likely treated with trimethoprim/sulfamethoxazole (TMP/SMX) compared to patients without SCVs (p = 0.0078).

### Patients with co-infecting pathogens

To assess the effect of co-infecting pathogens on lung function, we analyzed data of patients according to co-infection by important CF pathogens. Patients with *S*. *maltophilia* (n = 44) were more likely female (p = 0.0078), less likely nasal *S*. *aureus* carriers (p = 0.0003), more likely co-infected with SCVs (p<0.0001), experienced more exacerbations (p = 0.0165) and worse lung function of -3.72% FEV_1_% predicted throughout the study period (Fig 5, p = 0.0053, 95%CI -6.33 to -1.10). Patients with *A*. *fumigatus* (n = 60) were older (p<0.0001) and more likely co-infected by SCVs (p = 0.0001).

### IL-6 levels

**Fig 6:**
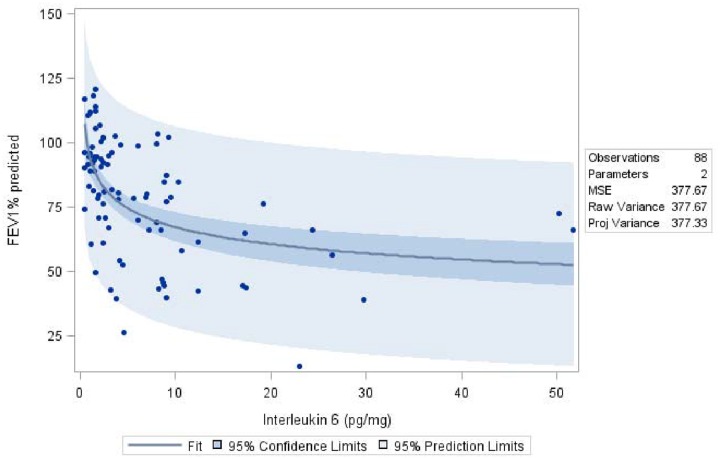
Higher IL-6 is associated with lower FEV1% predicted. Scatterplot of baseline measurements (n = 88) of all patients with blood samples provided at the first visit. The fit line shows the results of a non-linear regression model. Dark shaded areas show 95% confidence band, lighter shaded area shows 95% prediction band.

Since interleukin-6 levels have been shown to be helpful as a potential marker for severity of inflammation [14], we determined IL-6 in sera of the study patients. An inverse association between IL-6 levels and FEV_1_% predicted with worse lung function being associated with higher IL-6 at baseline (p<0.001, Fig 6). IL-6 levels were also associated with *S*. *aureus* density in sputa (p = 0.0016; difference of 0.6031 pg/ml in patients with high versus low *S*. *aureus* density), but not with bacterial density in throat or nasal cultures. Moreover, a significant increase in IL-6 levels in patients with exacerbations (p = 0.0411; difference of 0.2104 pg/ml), SCVs (p = 0.0209; difference 0.2287 pg/ml), *S*. *maltophilia* (p = 0.0195; difference 0.2622 pg/ml) or with *A*. *fumigatus* (p = 0.0496; 0.2256 pg/ml) was documented. In addition, significantly more CF patients with high bacterial loads displayed elevated IL-6 levels above the upper normal limit of 12 pg/ml [14,23,24] compared to patients with sputa containing low bacterial loads (p = 0.0142).

### Humoral response towards *S*. *aureus*

Recently, the height of *S*. *aureus* toxin-specific IgG-levels has been associated with the presence of *S*. *aureus* in airway specimens of CF patients, the severity of exacerbations and with FEV_1_% predicted [25]. To expand on these results, we measured IgG-levels against 44 different virulence factors of *S*. *aureus* (Supplemental Materials) in single serum samples of 182 CF patients and included the results in our analysis. Furthermore, IgG-levels of patients were compared to those of 53 healthy adult volunteers with persistent nasal *S*. *aureus* carriage. IgG-levels against 15 out of 44 analyzed antigens were significantly higher in patients compared to controls (p<0.001, Table 3). It is noteworthy that, IgG-levels against 22 virulence factors were significantly and positively associated with IL-6 levels in all CF patients and, in line with previous data, IgG-levels against 18 virulence factors were significantly and inversely associated with FEV_1_% predicted (S2 Table). A significant positive association between specific IgG-levels and nasal carriage, presence of SCVs and exacerbations for respectively 14, 16 and 7 antigens (S2 Table) was reported. Finally, no significant differences were found in IgG-levels of patients expectorating sputum with high and low bacterial loads (S2 Table).

**Table 3:** Significant specific IgG levels against *S*. *aureus* antigens in CF patients compared to healthy controls.

Antigen	Mean IgG level patients (± SE)^a^	Mean IgG level controls (± SE)^a^	p value^b^^,^^e^	Correlation coefficient of IgG and IL-6 levels^c^	p value^d^
Efb	4605 (±210)	2089 (±208)	< 0.0001	0.010	0.840
Glucosaminidase	14992 (±250)	11567 (±571)	< 0.0001	0.147	0.002
HlgB	13621 (±174)	9878 (±407)	< 0.0001	0.061	0.197
IsaA	13288 (±413)	10479 (±678)	0.0009	0.207	< 0.0001
IsdA	10701 (±250)	7638 (±429)	< 0.0001	0.304	< 0.0001
LukD	14176 (±248)	11485 (±464)	< 0.0001	0.14	0.003
LukE	14272 (±238)	11720 (±474)	< 0.0001	0.169	< 0.0001
LukF	4079 (±121)	2718 (±243)	< 0.0001	0.169	< 0.0001
LukS	14097 (±239)	7134 (±524)	< 0.0001	0.206	< 0.0001
LytM	6576 (±308)	3555 (±380)	0.0001	0.27	< 0.0001
Nuc	14405 (±271)	4752 (±493)	< 0.0001	0.0837	0.0795
SA0688	3063 (±259)	760 (±116)	< 0.0001	0.355	< 0.0001
SSL5	2589 (±128)	1616 (±111)	< 0.0001	-0.020	0.669
SSL9	8910 (±194)	7568 (±306)	0.0009	0.079	0.098
Wall teichoic acid	2491 (±124)	319 (±117)	< 0.0001	0.109	0.025

^a^Average IgG levels are given in mean fluorescence activity (MFI).

^b^p-values of difference between 190 patients and 53 healthy controls (Mann-Whitney U test)

^c^Spearman’s rank correlation coefficients were calculated for IgG- and IL-6 levels in all patients.

^d^p-values of correlation between IgG and IL-6

^e^adjusted p-values (Bonferroni correction)

## Discussion

Using a generalized linear mixed model our data only seem to support our main hypothesis stating that *S*. *aureus* density has an impact on lung function for patients who provided throat swabs since we could show that patients with higher bacterial density in throat cultures experienced a steeper lung function decline compared to patients with low bacterial density in these airway specimens, Fig 1. However, a closer look at our data showed that this effect was mostly carried by older patients, who provided throat cultures. The effect was lost, if only the group of patients younger than 20 years was analyzed, which is why these results should be interpreted carefully. Yet a trend of worse lung function of patients with high *S*. *aureus* density in sputa compared to patients with low *S*. *aureus* density was observed during the study period if cumulative clinical symptoms according to Fuchs were included in the linear mixed model analysis (p = 0.0661, S1 Fig).

With our inclusion and exclusion criteria a subgroup of patients was selected that differed from the German CF population by a preponderance of male patients (61.5%) and overrepresentation of non-p.Phe508del *CFTR* genotypes (27.7%). The fact that our study patients experienced a mean lung function decline of -1.2717 FEV_1_% predicted per years of age are consistent with German registry data that carriers of non-F508del genotypes become later in life colonized with *P*. *aeruginosa* than F508del compound heterozygotes and homozygotes and experience a milder course of the disease[26]. Nevertheless, our study patients still fit into the general CF-population with a lung function decline of 1 to 3 FEV_1_% points per year as previously described [27,28] and do therefore not differ in terms of severity of the clinical disease.

Recently, in a retrospective single center study Ahlgren et al. compared CF patients, who were colonized with *S*. *aureus* or *P*. *aeruginosa* only, or neither of these pathogens [29]. In their study, which also included more male patients (n = 55%), 24% of 84 patients were colonized by *S*. *aureus* [29]. In conjunction with our data this may suggest that male patients are more likely colonized by *S*. *aureus* only and are more likely resistant to colonization or infection caused by *P*. *aeruginosa*.

Several studies have shown that persistent nasal *S*. *aureus* carriers are at increased risk for severe *S*. *aureus* infections, but have a survival advantage during sepsis [13]. We thus determined the impact of nasal *S*. *aureus* carriage in CF patients on disease progression. Nasal carriage was higher than in other studies assessing nasal carriage as a risk factor for *S*. *aureus* infection [30,31] both in healthy persons [32] and in CF patients [11], but comparable to the study of Goerke et al. [11,12]. This is most likely attributable to the pre-selection on the basis of our inclusion criteria with the requirement of *S*. *aureus* persistence in patients’ airways.

During the study period the number of patients experiencing at least one pulmonary exacerbation was somewhat lower in our study compared to the study by Sanders et al. (31% versus 40%), who determined the association of frequency of exacerbations and subsequent lung function decline in more than 8.000 CF patients retrospectively [33]. This observation is most likely due to the fact that we excluded patients with chronic *P*. *aeruginosa* infection who have been shown to experience a more severe course of disease [34].

Our data thus clearly show that the culture of SCVs was independent of *P*. *aeruginosa* co-infection, but associated with advanced age, lower lung function and treatment with TMP/SMX at both base line and during the study period. Recently, Wolter et al. showed that the recovery of SCVs in airway specimens is independently associated with worse lung function in children [35]. Further studies also indicate that the detection of *S*. *aureus* SCVs was associated with persistence of *S*. *aureus* in airway specimens [19], advanced age [36], worse lung function [35–37] and co-infection with *P*. *aeruginosa* [35,36]. However, in our study we ruled out any impact of *P*. *aeruginosa* by excluding patients with persistent *P*. *aeruginosa* infection. In future analyses, it will be investigated if special geno- or phenotypes of *S*. *aureus* have an impact on the clinical status of the patients.

In our study, patients co-infected by *S*. *maltophilia* differed from patients without *S*. *maltophilia*. They were most likely female, not *S*. *aureus* nasal carriers, co-infected with *S*. *aureus* SCVs and experienced more exacerbations. While Goss et al. [38] did not find any impact of *S*. *maltophilia* on lung function, Waters et al. have recently shown that *S*. *maltophilia* represented an independent risk factor for pulmonary exacerbations [39], when patients were persistently colonized with *S*. *maltophilia*. Although we did not distinguish between *S*. *maltophilia* cultured once or persistently, co-infection by *S*. *maltophilia* clearly had a negative impact on the clinical status of our patients.

Also, patients co-infected by *A*. *fumigatus* significantly differed from patients without *A*. *fumigatus* with respect to age and prevalence of SCVs. Surprisingly, patients with *A*. *fumigatus* exhibited a better lung function (data not shown) compared to patients without the fungus. This is in contrast to the results reported by Amin et al. [40] showing worse lung function for 37 out of 230 CF patients with persistent *A*. *fumigatus* infection. This discrepancy may be due to the fact that we did not distinguish between intermittent and chronic infection. It may also be explained by a sampling effect as we primarily cultured *A*. *fumigatus* from a small number of mostly *A*. *fumigatus*-positive older patients.

Recently, Horsley et al. documented IL-6 as a key biomarker of inflammation in CF patients with exacerbations [14]. We found that IL-6 levels were significantly correlated with *S*. *aureus* density in sputa, and with the presence of exacerbations, SCVs, *S*. *maltophilia* or *A*. *fumigatus*. Thus, these results suggest that IL-6 is a highly sensitive marker for lung disease. IL-6 may potentially be used to identify patients, which might benefit from initiation of antibiotic therapy.

CF patients mounted higher IgG-levels against numerous virulence factors of *S*. *aureus* compared to healthy controls. Such an immune response against *S*. *aureus* is comparable to those of patients that exhibited *S*. *aureus* bacteremia or osteomyelitis [20]. The fact that the adaptive immune system produces specific antibodies against *S*. *aureus* underlines that persistently colonized CF patients are immunologically challenged by *S*. *aureus* although these specific antibodies cannot resolve *S*. *aureus* persistence. While no significant association between bacterial density in airway specimens of CF patients and IgG-levels was reported, we found a significant association of many specific IgG-values with IL-6-levels, worse lung function, nasal carriage and the culture of SCVs. The latter confirms and expands on another recent study that demonstrated a significant inverse association between *S*. *aureus* toxin-specific IgG-levels and lung function in CF patients [25]. In contrast to our results, this previous study also showed a significant association between the culture of *S*. *aureus* in airway specimens and IgG-levels, albeit bacterial density was not quantified as it was done in our study.

There are several limitations to our study. First, our inclusion and exclusion criteria selected a sub-group of CF patients, which do not reflect the entire CF population. However, lung function data of our patients were comparable to other studies [27,41]. Our data should thus provide meaningful results for the general CF population. Second, our selected patient group did experience somewhat less exacerbations compared to groups with *P*. *aeruginosa* infection, which makes it more difficult to detect significant clinical differences between groups using exacerbation as an outcome measure. Third, the technique of obtaining throat swabs in the different centres by different nurses or physicians could have influenced the microbiological results of the throat swab cultures. Fourth, it seems that our study was underpowered to show significant results for the association between *S*. *aureus* density and worse lung function of patients, who were able to expectorate sputum. Since *S*. *aureus* is the most prevalent CF-related pathogen in children, we included patients in our study who were not able to expectorate sputum. We hypothesized that the determination of bacterial densities would be of equal value for throat swabs and sputa. Our patient number calculation was based on this hypothesis. However, there was no association between bacterial density and lung function for patients with throat swabs, but a trend for patients with high bacterial density in sputa if cumulative clinical symptoms for exacerbation were included in the analysis (0.0661, S1 Fig).

To conclude, our results suggest that the presence of exacerbations, non-nasal *S*. *aureus* carriage, female gender, the presence of *S*. *aureus* SCVs, co-infection with *S*. *maltophilia* or specific anti-staphylococcal IgG- and IL-6 levels are independent risk factors for worse lung function in CF patients with persistent *S*. *aureus* infection. Our findings may help to identify *S*. *aureus* patients at risk for more severe airway infections resulting in worse lung function and will be of value for designing future prospective studies to guide antibiotic therapy in patients chronically infected with *S*. *aureus*.

## Supporting Information

S1 Fig FEV1% predicted and bacterial density in sputa. (PDF)

S1 Supporting Material (DOCX)

S1 Table Clinical characteristics of patients at baseline. (DOCX)

S2 Table Specific antistaphylococcal IgG levels associated with clinical condition. (DOCX)
