# Supplementary material for: Factors Associated with Worse Lung Function in Cystic Fibrosis Patients with Persistent Staphylococcus aureus
Source: PLoS One. 2016 Nov 18;11(11):e0166220. doi: 10.1371/journal.pone.0166220 (PMC5115705; doi:10.1371/journal.pone.0166220)
Supplement: S1 Fig — (PDF) [file pone.0166220.s001.pdf]

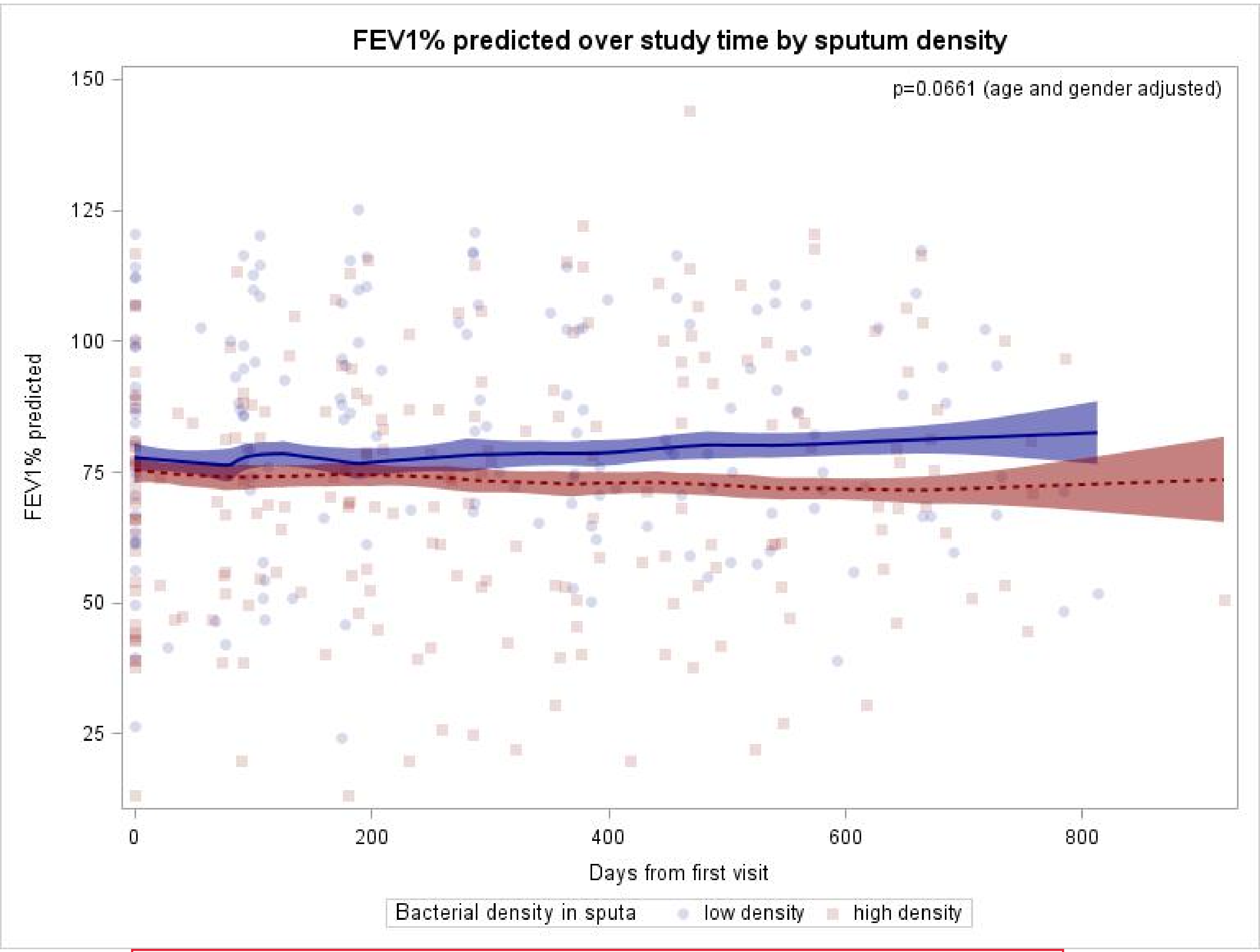

Figure legend for Figure S1. FEV1% predicted over study time by sputum density.

The figure shows observed FEV_1% predicted measurements over study time (days from first visit). Lines represent the LOESS-fit including 95% confidence intervals of the predicted values of the generalized linear mixed model. Patients with high bacterial density in sputum are indicated by red squares, patients with low bacterial density in sputum by blue circles, the fitted model prediction is coloured accordingly.
