# Supplementary material for: Factors Associated with Worse Lung Function in Cystic Fibrosis Patients with Persistent Staphylococcus aureus
Source: PLoS One. 2016 Nov 18;11(11):e0166220. doi: 10.1371/journal.pone.0166220 (PMC5115705; doi:10.1371/journal.pone.0166220)
Supplement: S1 Supporting Material — (DOCX) [file pone.0166220.s002.docx]

**S1-Supporting Material**

**Factors associated with lung function decline in cystic fibrosis patients with persistent *Staphylococcus aureus***

Sibylle Junge^1*^, Dennis Görlich^2*^, Martijn den Reijer^3^, Bärbel Wiedemann^4^, Burkhard Tümmler^5^, Helmut Ellemunter^6^, Angelika Dübbers^7^, Peter Küster^8^, Manfred Ballmann^9^, Cordula Koerner-Rettberg^9^, Uwe Mellies^10^, Eberhardt Heuer^11^, Wolfgang Sextro^11^, Jochen G. Mainz^12^, Jutta Hammermann^13^, Joachim Riethmüller^14^, Ute Graepler-Mainka^14^, Doris Staab^15^, Bettina Wollschläger^16^, Rüdiger Szczepanski^17^, Antje Schuster^18^, Friedrich-Karl Tegtmeyer^19^, Sivagurunathan Sutharsan^20^, Alexandra Wald^21^, Jerzy-Roch Nofer^22^, Willem van Wamel^3^, Karsten Becker^23^, Georg Peters^23^, Barbara C. Kahl^23^

**IL-6 levels**

The assay was characterized by intra-assay precisions of 4.2%, 1.6% and 2.0% at low, medium and high analyte levels, respectively. The minimum detectable dose of IL-6 was less than 0.7 pg/mL.The cut-off value for IL-6 (12 pg/mL) corresponds to the highest IL-6 level observed in a control population of 40 apparently healthy individuals and is very close to the cut-off value of 15 pg/mL, which has been suggested in previously published studies [3, 4].

**Bacterial antigens for the detection of *S. aureus*-specific IgG**

The following 55 antigens were used to detect *S. aureus*-specific antibodiesby coupling to xMAP® beads (Luminex Corporation, Austin, TX, USA): Protein secretion system ESX-1-associated factors EsxA and B; Nuclease (Nuc); peptidoglycan hydrolase LytM; immunodominant antigen A (IsaA); glucosaminidase; lipase; peptidoglycan (PG); wall teichoic acid (WTA); foldase-protein PrsA; clumping factor A and B (ClfA and ClfB); SD-repeat containing proteins D and E (SdrD and SdrE); iron-responsive surface determinants A and H (IsdA and IsdH); fibronectin-binding proteins A and B (FnbpA and FnbpB); extracellular fibrinogen-binding protein (Efb); *S. aureus* surface protein G (SasG); staphylococcal complement inhibitor (SCIN); chemotaxis inhibitory protein of *S. aureus* (CHIPS); formyl peptide receptor-like inhibitory protein (FLIPr); alpha toxin; gamma-hemolysin B (HlgB); leukocidins D, E, F and S (LukD, LukE, LukF and LukS); staphylococcal enterotoxins A-E, G-I, M-O, Q, R (SEA–SEE, SEG-SEI, SEM-SEO, SEQ, SER); exfoliative toxins A and B (ETA and ETB); toxic shock syndrome toxin 1 (TSST-1); staphylococcal superantigen-like proteins 1, 3, 5, 9, 10 and 11 (SSL1, SSL3, SSL5, SSL9, SSL10 and SSL11) and hypothetical proteins SA0486 and SA0688.

IgG levels against 44 antigens could be evaluated, see Table S1.

**REFERENCES**

1. Fuchs HJ, Borowitz DS, Christiansen DH, et al. Effect of aerosolized recombinant human DNase on exacerbations of respiratory symptoms and on pulmonary function in patients with cystic fibrosis. The Pulmozyme Study Group. N Engl J Med **1994**; 331:637-642.

2. Kahl B, Herrmann M, Everding AS, et al. Persistent infection with small colony variant strains of *Staphylococcus aureus* in patients with cystic fibrosis. J Infect Dis **1998**; 177:1023-1029.

3. Gaini S, Koldkjaer OG, Pedersen C, Pedersen SS. Procalcitonin, lipopolysaccharide-binding protein, interleukin-6 and C-reactive protein in community-acquired infections and sepsis: a prospective study. Crit Care **2006**; 10:R53.

4. Mardi D, Fwity B, Lobmann R, Ambrosch A. Mean cell volume of neutrophils and monocytes compared with C-reactive protein, interleukin-6 and white blood cell count for prediction of sepsis and nonsystemic bacterial infections. Int J Lab Hematol **2010**; 32:410-418.
