# Supplementary material for: Factors Associated with Worse Lung Function in Cystic Fibrosis Patients with Persistent Staphylococcus aureus
Source: PLoS One. 2016 Nov 18;11(11):e0166220. doi: 10.1371/journal.pone.0166220 (PMC5115705; doi:10.1371/journal.pone.0166220)
Supplement: S1 Table — (DOCX) [file pone.0166220.s003.docx]

**S1 Table. Clinical patients’ characteristics at baseline**

| **Characteristics** | **all patients^*^** | **nasal carriers** | **non-carriers** | **p-values** | **with**  **exacerbation**** | **without exacerbation** | **p-values** | **with SCVs**  **ever** | **without SCVs** | **p-values** |
| --- | --- | --- | --- | --- | --- | --- | --- | --- | --- | --- |
| **# of patients** | 195 | 122 (65%)^†^ | 66 (35%) |  | 60 (31%) | 135 (69%) |  | 84 (43%) | 111 (57%) |  |
| **Age (years)**  **Male** | 16  (6-42)  120 (61.54%) | 14.083  (6.02-41.58)  86 (70.5%) | 13.33  (5.82-40.7)  30 (45.5%^) | 0.45^‡^  0.00075^§^ | 14.95  (7.23-36.37)  33 (55%) | 13.65  (5.82-41.7)  87 (64.44%) | 0.5182^‡^  0.2109^§^ | 15.41  (5.82-41.58)  48 (57.14%) | 13.03  (6.02-40.53)  72 (64.86%) | 0.0066^‡^  0.2724^§^ |
| **Ex. panc.suff.^‖^** | 22 (11%) | 17 | 5 | 0.201^§^ | 3 (5.08%) | 19 (14.18%) | 0.067^§^ | 9 (10.8%) | 13 (12.04%) | 0.7978^§^ |
| **FEV_1_**  **(% predicted)** | 84.07  (13.18-120.57) | 86.96  (13.18-120.57) | 80.92  (26.35-118.05) | 0.096^‡^ | 81.64  (26.35-116.82) | 86.27  (13.18-120.57) | 0.1941^‡^ | 79.48  (13.18-120.57) | 88.56  (30.56-115.19) | 0.0133^‡^ |
| **BMI-Quantils** | 25%  (0.068%-100%) | 28%  (1%-100%) | 23%  (0.068%-81%) | ns^‡^ | 26.77%  (1.7%-97.29%) | 24.93%  (0.068%-99.79%) | 0.7740^‡^ | 23%  (0.068%-92%) | 27%  (4%-100%) | 0.3166^‡^ |
| **Genotype^††^**  **F508del homo^‡‡^**  **F508del hetero^§§^**  **others** | 173  85 (49%)  40 (23.2%)  48 (27.7%) | 52 (48.1%)  25 (23.1%)  31 (28.7%) | 28 (48.3%)  13 (22.4%)  17 (29.3%) | 0.993^§^ | 27 (54%)  14 (28%)  9 (18%) | 58 (47.15%)  26 (21.14%)  39 (31.71%) | 0.1764^§^ | 35 (49.3%)  11 (15.5%)  25 (35.2%) | 49 (49%)  28 (28%)  23 (23%) | 0.079^§^ |

| **Characteristics** | **With**  ***S. maltophilia*** | **Without**  ***S. maltophilia*** | **p-values** | **With**  ***A. fumigatus*** | **Without**  ***A. fumigatus*** | **p-values** |
| --- | --- | --- | --- | --- | --- | --- |
| **# of patients** | 44 | 151 |  | 60 (31%) | 135 (69%) |  |
| **Age (years)**  **Male** | 15.78  (7.33-36.37)  20 (45.45%) | 13.51  (5.82-41.58)  100 (66.23%) | 0.0847^‡^  0.0127^§^ | 18.10  (8.99-41.58)  34 (56.67%) | 12.85  (5.82-40.70)  86 (63.7%) | <0.0001^‡^  0.3512^§^ |
| **Ex. panc.suff.^‖^** | 2 (4.55%) | 20 (13.61%) | 0.0986^§^ | 7 (11.86%) | 15 (11.36) | 0.9202^§^ |
| **FEV_1_**  **(% predicted)** | 80.05  (38.96-120.57) | 85.89  (13.18-118.05) | 0.2071^‡^ | 80.41  (37.56-120.57) | 85.60  (13.18-118.05) | 0.2698^‡^ |
| **BMI-Quantils** | 23%  (5%-92%) | 25%  (0.068%-100%) | 0.8861^‡^ | 23%  (1%-97%) | 27%  (0.068%-100%) | 0.2332^‡^ |
| **Genotype^††^**  **F508del homo^‡‡^**  **F508del hetero^§§^**  **others** | 171  20 (52.63%)  8 (21.05%)  10 (26.32%) | 64 (48.12%)  31 (23.31%)  38 (28.57%) | 0.8861^§^ | 171  26 (49.06%)  12 (22.64%)  15 (28.3%) | 58 (49.15%)  27 (22.88%)  33 (27.97%) | 0.9988^§^ |

^*^Age, FEV1, BMI and exacerbation numbersare reported as median(range), respectively. Exocrine pancreatic sufficiency and genotype are reported as absolute frequencies (relative frequencies).

^†^patients with only one or two nasal swabs were excluded from the analysis (n=7)

^‡^Mann-Whitney U-test

^§^Chi-Quadrat test

^‖^exocrine pancreatic sufficiency

^**^percentage of exacerbations as indicated in CRFs calculated in percentage for every patient with the range

^††^*Cftr* genotype of patients available

^‡‡^F508del homozygous

^§§^F508del heterozygous
