# Supplementary material for: Factors Associated with Worse Lung Function in Cystic Fibrosis Patients with Persistent Staphylococcus aureus
Source: PLoS One. 2016 Nov 18;11(11):e0166220. doi: 10.1371/journal.pone.0166220 (PMC5115705; doi:10.1371/journal.pone.0166220)
Supplement: S2 Table — (DOCX) [file pone.0166220.s004.docx]

| **Antigen** | **Mean IgG level all patients (± SE)^1^** | **Mean IgG level controls (± SE)^1^** | **p value^2,3^** | **Mean IgG level high bacterial density (± SE)^4^** | **Mean IgG level low bacterial density (± SE)^5^** | **p value^2, 3^** | **Estimated effect on IL-6 levels^6^** | **p value^3^** | **Estimated effect on FEV1%^7^** | **p value^3^** | **OR nasal carrier status^9^** | **p value^3^** | **OR SCV presence^9^** | **p value^3^** | **OR exacerbation^10^** | **p value^3^** |
| --- | --- | --- | --- | --- | --- | --- | --- | --- | --- | --- | --- | --- | --- | --- | --- | --- |
| Alpha toxin | 14405 (±271) | 13902 (±511) | 0.2653 | 14430 (±345) | 14493 (±282) | 0.8085 | -1.33E-6 | 0,9226 | -0,00013 | 0,6506 | **1,010** | **0,0008** | 0,996 | 0,2112 | 0,995 | 0,0993 |
| CHIPS | 11492 (±220) | 11019 (±349) | 0.104 | 11546 (±319) | 11617 (±254) | 0.6691 | 0,000019 | 0,2173 | **-0,00073** | **0,0185** | 1,003 | 0,4282 | 1,000 | 0,9999 | 1,002 | 0,4990 |
| ClfA | 5168 (±237) | 4302 (±436) | 0.081 | 5617 (±322) | 5222 (±262) | 0.3415 | -0,00002 | 0,1179 | 0,000514 | 0,1104 | 1,009 | 0,0174 | **1,011** | **0,0011** | 1,001 | 0,6727 |
| ClfB | 4587 (±222) | 4092 (±338) | 0.3809 | 5221 (±321) | 4562 (±214) | 0.2075 | 3.208E-6 | 0,8616 | **0,002079** | **<.0001** | 1,005 | 0,2808 | 0,995 | 0,2333 | 0,995 | 0,2456 |
| Efb | 4605 (±210) | 2089 (±207) | < 0.0001 | 4610 (±290) | 4698 (±236) | 0.6336 | 0,000034 | 0,0650 | -0,00071 | 0,0531 | 0,995 | 0,2490 | 1,001 | 0,7260 | 1,000 | 0,9118 |
| ETA | 3999 (±447) | 2043 (±424) | 0.1180 | 3868 (±532) | 3884 (±421) | 0.7338 | **0,000026** | **0,0173** | **-0,00061** | **0,0033** | 1,004 | 0,0741 | 1,000 | 0,9101 | 1,001 | 0,7047 |
| ETB | 613 (±113) | 320 (±94) | 0.0032 | 532 (±135) | 341 (±48) | 0.7725 | **0,000161** | **0,0014** | **-0,00700** | **<.0001** | 1,032 | 0,0743 | 1,014 | 0,2320 | 1,002 | 0,8706 |
| FlipR | 5708 (±251) | 4834 (±400) | 0.0784 | 6203 (±333) | 5487 (±277) | 0.0931 | 0,000020 | 0,2025 | -0,00040 | 0,1852 | 1,009 | 0,0149 | 0,999 | 0,8526 | 0,998 | 0,5126 |
| FnbpA | 2922 (±201) | 2534 (±300) | 0.6167 | 3116 (±252) | 2979 (±194) | 0.1881 | 0,000031 | 0,1775 | 0,000222 | 0,6310 | 1,010 | 0,0723 | 1,010 | 0,0723 | 1,014 | 0,0060 |
| FnbpB | 967 (±79) | 1327 (±215) | 0.1306 | 1286 (±144) | 1036 (±71) | 0.2802 | 0,000053 | 0,3988 | -0,00199 | 0,1150 | 0,991 | 0,5160 | 1,010 | 0,4724 | 0,964 | 0,0216 |
| Glucosaminidase | 14992 (±250) | 11567 (±571) | < 0.0001 | 15507 (±308) | 15174 (±256) | 0.2996 | **0,000061** | **<.0001** | **-0,00090** | **0,0014** | 1,003 | 0,3082 | **1,010** | **0,0047** | 1,004 | 0,2303 |
| HlgB | 13621 (±174) | 9878 (±407) | < 0.0001 | 13705 (±235) | 13624 (±206) | 0.7169 | **0,000063** | **0,0027** | **-0,00109** | **0,0088** | 1,003 | 0,4992 | 1,010 | 0,0290 | 1,003 | 0,5584 |
| IsaA | 13288 (±413) | 10479 (±678) | 0.0009 | 13963 (±595) | 12654 (±466) | 0.0524 | **0,000032** | **0,0003** | **-0,00064** | **0,0002** | 1,004 | 0,0300 | 1,001 | 0,6861 | 1,002 | 0,2518 |
| IsdA | 10701 (±250) | 7638 (±428) | < 0.0001 | 11133 (±283) | 10871 (±283) | 0.0732 | **0,000085** | **<.0001** | **-0,00152** | **<.0001** | **1,010** | **0,0028** | **1,009** | **0,0083** | 1,000 | 0,9086 |
| IsdH | 3220 (±238) | 3643 (±380) | 0.0910 | 3281 (±296) | 2816 (±207) | 0.0981 | **0,000063** | **0,0010** | -0,00039 | 0,3074 | 0,998 | 0,7124 | 1,003 | 0,4299 | 1,002 | 0,6094 |
| Lipase | 9979 (±317) | 8237 (±629) | 0.0104 | 10318 (±439) | 9266 (±361) | 0.0761 | **0,000032** | **0,0055** | -0,00025 | 0,2749 | 1,002 | 0,5068 | 1,003 | 0,2340 | 1,003 | 0,2450 |
| LukD | 14176 (±248) | 11485 (±463) | < 0.0001 | 14598 (±314) | 13916 (±292) | 0.0789 | **0,000052** | **0,0003** | **-0,00128** | **<.0001** | 1,003 | 0,4125 | 1,008 | 0,0195 | 1,001 | 0,7464 |
| LukE | 14272 (±238) | 11720 (±473) | < 0.0001 | 14752 (±305) | 13998 (±287) | 0.0819 | **0,000067** | **<.0001** | **-0,00136** | **<.0001** | 1,000 | 0,9943 | **1,012** | **0,0012** | 1,007 | 0,0323 |
| LukF | 4079 (±133) | 2718 (±243) | < 0.0001 | 4526 (±200) | 4144 (±147) | 0.0892 | **0,000100** | **0,0002** | **-0,00305** | **<.0001** | 0,998 | 0,7741 | 1,003 | 0,5710 | 1,013 | 0,0254 |
| LukS | 14097 (±133) | 7134 (±524) | < 0.0001 | 11068 (±396) | 10586 (±295) | 0.1873 | **0,000064** | **<.0001** | **-0,00169** | **<.0001** | 0,995 | 0,0700 | **1,009** | **0,0015** | 1,004 | 0,1382 |
| LytM | 6576 (±367) | 3555 (±380) | 0.0001 | 7059 (±492) | 6248 (±382) | 0.0542 | **0,000054** | **<.0001** | **-0,00069** | **0,0014** | 0,999 | 0,7773 | **1,007** | **0,0030** | 1,005 | 0,0361 |
| Nuc | 14405 (±271) | 4752 (±492) | < 0.0001 | 10852 (±503) | 9881 (±394) | 0.0707 | 0,000022 | 0,0242 | -0,00021 | 0,2689 | **1,007** | **0,0011** | 1,004 | 0,0409 | 0,997 | 0,1049 |
| SA0486 | 446 (±26) | 341 (±42) | 0.0397 | 450 (±43) | 488 (±41) | 0.5357 | 4.081E-6 | 0,9519 | 0,000285 | 0,8300 | 0,971 | 0,1795 | 0,995 | 0,7294 | 1,004 | 0,7859 |
| SA0688 | 3063 (±259) | 759 (±116) | < 0.0001 | 3449 (±346) | 3177 (±220) | 0.1731 | **0,000113** | **<.0001** | **-0,00217** | **<.0001** | 1,000 | 0,9754 | 1,008 | 0,0314 | 1,007 | 0,0570 |
| SasG | 569 (±70) | 636 (±132) | 0.1847 | 623 (±95) | 388 (±50) | 0.1852 | 0,000016 | 0,7673 | 0,001484 | 0,1590 | 1,002 | 0,8445 | 1,049 | 0,0157 | 1,006 | 0,5492 |
| SCIN | 10872 (±247) | 9657 (±388) | 0.0043 | 11226 (±321) | 10470 (±277) | 0.0655 | **0,000039** | **0,0060** | -0,00063 | 0,0241 | **1,009** | **0,0032** | 1,006 | 0,0684 | 0,995 | 0,1129 |
| SdrD | 1084 (±80) | 694 (±76) | 0.0507 | 1108 (±105) | 937 (±65) | 0.2933 | **0,000138** | **0,0160** | -0,00145 | 0,1950 | 0,973 | 0,0260 | 1,029 | 0,0232 | 0,985 | 0,2246 |
| SdrE | 3139 (±201) | 1992 (±221) | 0.0253 | 3513 (±275) | 3351 (±199) | 0.2916 | **0,000068** | **0,0010** | **-0,00179** | **<.0001** | 1,003 | 0,4736 | **1,013** | **0,0059** | 1,000 | 0,9768 |
| SEA | 3835 (±320) | 3613 (±425) | 0.2544 | 4398 (±437) | 3539 (±321) | 0.1111 | -0,00001 | 0,3454 | -0,00007 | 0,7599 | 1,005 | 0,1021 | 1,004 | 0,0935 | 0,997 | 0,2508 |
| SEC | 7390 (±441) | 8714 (±809) | 0.1350 | 6901 (±581) | 6878 (±473) | 0.9446 | -9.9E-6 | 0,2951 | 0,000269 | 0,1366 | 1,000 | 0,9743 | 1,000 | 0,9411 | 0,999 | 0,6049 |
| SED | 1106 (±118) | 1292 (±268) | 0.0498 | 1150 (±153) | 1115 (±135) | 0.1178 | 0,000018 | 0,6231 | 0,001490 | 0,0377 | 1,010 | 0,2704 | 0,998 | 0,7832 | 0,999 | 0,8861 |
| SEE | 1333 (±183) | 879 (±136) | 0.8113 | 1416 (±214) | 1039 (±142) | 0.1334 | -6.43E-7 | 0,9820 | -0,00057 | 0,2779 | 1,014 | 0,0608 | 1,015 | 0,0193 | 0,997 | 0,6557 |
| SEG | 2047 (±184) | 1225 (±270) | 0.0544 | 1941 (±190) | 1790 (±190) | 0.4405 | **0,000091** | **0,0005** | -0,00108 | 0,0264 | **1,029** | **0,0003** | 1,005 | 0,3829 | 0,998 | 0,7464 |
| SEH | 2290 (±291) | 2174 (±359) | 0.0113 | 2128 (±382) | 1950 (±281) | 0.9851 | -0,00001 | 0,3834 | -0,00047 | 0,0986 | **0,991** | **0,0039** | **1,009** | **0,0071** | 1,007 | 0,0249 |
| SEO | 368 (±40) | 400 (±51) | 0.0266 | 476 (±79) | 394 (±48) | 0.5521 | 0,000185 | 0,0452 | 0,001048 | 0,5874 | 1,094 | 0,0135 | 0,991 | 0,6676 | 0,944 | 0,0436 |
| SER | 1548 (±243) | 2001 (±580) | 0.3882 | 1755 (±377) | 1436 (±264) | 0.2225 | 0,000024 | 0,2410 | 0,000166 | 0,6522 | 1,007 | 0,1954 | 0,999 | 0,7642 | 1,007 | 0,0955 |
| SSL1 | 6856 (±410) | 5684 (±683) | 0.2735 | 7623 (±542) | 6359 (±424) | 0.0571 | 0,000021 | 0,0277 | -0,00029 | 0,1199 | **1,009** | **0,0002** | 1,001 | 0,7920 | 1,002 | 0,2828 |
| SSL3 | 5561 (±216) | 5123 (±313) | 0.4604 | 5737 (±285) | 5621 (±245) | 0.5586 | 3.401E-6 | 0,8398 | -0,00051 | 0,1235 | 0,996 | 0,2676 | 0,995 | 0,1844 | 0,988 | 0,0017 |
| SSL5 | 2589 (±128) | 1616 (±111) | < 0.0001 | 2639 (±170) | 2575 (±139) | 0.8226 | 0,000023 | 0,4368 | -0,00015 | 0,7957 | 1,013 | 0,0480 | **0,981** | **0,0052** | 0,997 | 0,5820 |
| SSL9 | 8910 (±194) | 7568 (±305) | 0.0009 | 9114 (±252) | 8917 (±235) | 0.5563 | 0,000034 | 0,0620 | 0,000681 | 0,0570 | 1,002 | 0,5567 | 1,000 | 0,9684 | 0,997 | 0,3912 |
| SSL10 | 8754 (±306) | 9870 (±552) | 0.0869 | 9107 (±420) | 8520 (±336) | 0.2516 | 0,000020 | 0,1071 | 0,000239 | 0,3165 | 0,999 | 0,6832 | 0,996 | 0,1613 | 1,000 | 0,9705 |
| SSL11 | 3127 (±255) | 2698 (±347) | 0.8779 | 3310 (±334) | 2969 (±267) | 0.0839 | 3.79E-6 | 0,8488 | **-0,00154** | **0,0001** | 1,006 | 0,2167 | 0,994 | 0,1617 | 0,992 | 0,0788 |
| TSST1 | 7981 (±487) | 9076 (±646) | 0.499 | 7585 (±634) | 7602 (±545) | 0.6607 | -7.19E-6 | 0,3762 | 0,000148 | 0,3554 | 1,004 | 0,0283 | 0,999 | 0,4654 | 0,998 | 0,1558 |
| Wall teichoic acid | 2491 (±124) | 319 (±16) | < 0.0001 | 2182 (±119) | 2038 (±119) | 0.2623 | **0,000070** | **0,0147** | **-0,00228** | **<.0001** | 1,008 | 0,2084 | 1,006 | 0,3328 | 1,012 | 0,0588 |

^1^significant difference of results between 182 patients and 53 healthy controls

^2^p values of difference between patient and controls groups (Mann-Whitney U test)

^3^adjusted p-values (Bonferroni correction)

^4^Mean IgG levels of 228 cases expectorating sputum with high bacterial density

^5^Mean IgG levels of 166 cases expectorating sputum with low bacterial density

^6^IgG levels are modelled as continuous factors. Estimated effects are interpreted as mean change in IL6 per 1 unit of IgG levels. Significant effects are marked bold.

^7^IgG levels are modelled as continuous factors. Estimated effects are therefore interpreted as mean change in FEV1% predicted per 1 unit of IgG levels. Significant effects are marked bold.

^8^Odds ratios (OR) are interpreted as factor by which the risk to be a nasal carrier is changed per 100 units of IgG levels. Significant OR are marked bold.

^9^Odds ratios (OR) are interpreted as factor by which the risk to ever be SCV positive is changed per 100 units of IgG levels. Significant OR are marked bold.

^10^Odds ratios (OR) are interpreted as factor by which the risk to ever experience an exacerbation is changed per 100 units of IgG levels. Significant OR are marked bold.
